# Direct RNA Sequencing Reveals SARS-CoV-2 m6A Sites and Possible Differential DRACH Motif Methylation among Variants

**DOI:** 10.3390/v13112108

**Published:** 2021-10-20

**Authors:** João H. C. Campos, Juliana T. Maricato, Carla T. Braconi, Fernando Antoneli, Luiz Mario R. Janini, Marcelo R. S. Briones

**Affiliations:** 1 Center for Medical Bioinformatics, Escola Paulista de Medicina, Federal University of São Paulo (UNIFESP), São Paulo 04039032, Brazil; joao.heima@gmail.com (J.H.C.C.); fernando.antoneli@unifesp.br (F.A.); 2 Department of Microbiology, Immunology and Parasitology, Escola Paulista de Medicina, Federal University of São Paulo (UNIFESP), São Paulo 04023062, Brazil; juliana.maricato@unifesp.br (J.T.M.); ctbsantos@unifesp.br (C.T.B.); janini@unifesp.br (L.M.R.J.)

**Keywords:** SARS-CoV-2, COVID-19, m6A, direct RNA sequencing, RNA methylation, Epitranscriptomics

## Abstract

The causative agent of COVID-19 pandemic, SARS-CoV-2, has a 29,903 bases positive-sense single-stranded RNA genome. RNAs exhibit about 150 modified bases that are essential for proper function. Among internal modified bases, the *N^6^*-methyladenosine, or m6A, is the most frequent, and is implicated in SARS-CoV-2 immune response evasion. Although the SARS-CoV-2 genome is RNA, almost all genomes sequenced thus far are, in fact, reverse transcribed complementary DNAs. This process reduces the true complexity of these viral genomes because the incorporation of dNTPs hides RNA base modifications. Here, we present an initial exploration of Nanopore direct RNA sequencing to assess the m6A residues in the SARS-CoV-2 sequences of ORF3a, E, M, ORF6, ORF7a, ORF7b, ORF8, N, ORF10 and the 3′-untranslated region. We identified fifteen m6A methylated positions, of which, six are in ORF N. Additionally, because m6A is associated with the DRACH motif, we compared its distribution in major SARS-CoV-2 variants. Although DRACH is highly conserved among variants, we show that variants Beta and Eta have a fourth position C > U change in DRACH at 28,884b that could affect methylation. This is the first report of direct RNA sequencing of a Brazilian SARS-CoV-2 sample coupled with the identification of modified bases.

## 1. Introduction

RNA viruses are causative agents of major human transmissible diseases such as influenza, poliomyelitis, measles and COVID-19 [1,2,3,4,5]. In the Baltimore classification, viruses with RNA genomes comprise groups III, IV, V and VI, while DNA viruses are in groups I, II and VII [6,7]. COVID-19 is a highly contagious viral disease with severe respiratory, inflammatory and thrombotic manifestations [8,9]. The COVID-19 pandemic is caused by a Beta coronavirus, SARS-CoV-2, included in Group IV of the Baltimore classification [10,11]. In SARS-CoV-2, the RNAs serve as information storage when packaged into the viral particle and as mRNAs for viral protein synthesis upon infection of mammalian cells [12,13].

The SARS-CoV-2 genome consists of a positive-sense single-stranded strand RNA with 29,903 bases [13]. There are approximately 150 different base modifications in all RNA species, and these modified bases are essential for proper translation, splicing and RNA metabolism [14]. Among these modified bases, the *N^6^*-methyladenosine (m6A) is the most frequent internal base modification, and is found in viruses with exclusive cytoplasm replication, such as Zika Virus, Dengue virus and Hepatitis C virus [15,16]. The methylated base m6A is implicated in SARS-CoV-2’s evasion of the host immune response because the methylated viral RNA does not interact with the host protein RIG-I (retinoic acid-inducible gene I) responsible for the type-1 interferon (IFN1) response, an activator of immune pathways [14,17,18]. The viral RNA is m6A methylated by the host’s methylases METTL3, METTL14, WATAP and KIAA1429, called “writers”, and demethylated by FTO and ALKBH5, called “erasers”, which normally demethylate the host’s RNAs [19]. Knockdown of METTL3 significantly reduces the SARS-CoV-2 methylation and blocks the viral mechanism of RIG-I binding inhibition [17].

Almost all the SARS-CoV-2 genomes sequenced thus far are reverse transcribed complementary DNAs (cDNAs), although the genome is, in fact, RNA [20]. Reverse transcription provides a fast, practical, PCR prone method for sequencing the SARS-CoV-2 genome. However, it reduces the true complexity of these viral genomes. The incorporation of dNTPs in the first strand cDNA chain makes the RNA base modifications, present in the RNA template chain, mostly indistinguishable from unmodified bases and sequencing errors [21]. RNA modified bases are critical for proper biological function and are involved in several diseases, encompassing the field of Epitranscriptomics Medicine [19]. Although different technologies have been used for the identification of modified bases in mRNAs and viral RNAs, they require substantial quantities of material that precludes single-cell analysis and low abundance samples. Additionally, the antibody-dependent methylation analysis does not provide nucleotide level accuracy of modified bases [14,17,18,22].

Oxford Nanopore Technology (ONT) has been used for SARS-CoV-2 whole-genome cDNA sequencing [23]. Additionally, this same technology has been used for direct RNA sequencing of SARS-CoV-2 [24,25,26,27]. The major advantage of ONT direct RNA sequencing over cDNA sequencing is the identification of modified bases. Two previous studies on SARS-CoV-2 direct RNA sequencing did not couple the genetic analysis with base modification identification, while a third study detected the 5mC methylation using this technology [24,26,27]. A fourth study detected sgRNAs in supernatants of Calu-3 cells (human lung epithelial) using direct RNA sequencing and detected modified bases but not specified by type [25].

In the present study, we assessed the potential of the Nanopore direct RNA sequencing for the identification of m6A residues, at nucleotide level resolution, in the SARS-CoV-2 genome. For this, we analyzed direct RNA sequencing reads of open reading frames (ORFs) 3a, E, M, 6, 7a, 7b, 8, N, 10 and the 3′-untranslated region [22]. In addition, since m6A is associated with the DRACH motif (D = G/A/U, R = G/A, H = A/U/C), we compared the DRACH distribution in major SARS-CoV-2 variants to verify if potential variant-specific alterations in m6A methylation patterns occur in SARS-CoV-2 evolution [28].

## 2. Materials and Methods

### 2.1. Cell Cultures and SARS-CoV-2 Infection

All procedures for viral isolation and initial passages were performed in a biosafety level 3 laboratory (BLS3), in accordance with WHO recommendations and under the laboratory biosafety guidance required for SARS-CoV-2 at the BLS3 facilities at the Federal University of São Paulo. SARS-CoV-2 stock was kindly provided by Prof. José Luiz Proença-Módena (University of Campinas—UNICAMP, SP, Brazil).

For SARS-CoV-2 infection, the Vero E6 cell line (ATCC^®^ CRL-1586™) was maintained in Minimum Essential Medium (MEM; Gibco) supplemented with 10% fetal bovine serum (FBS) (Gibco) and 1% penicillin/streptomycin (Gibco). Vero E6 cells were kept in a humidified 37 °C incubator with 5% CO_2_. After reaching an 80% confluent monolayer, cells were seeded in 24-well plates at a density of 5 × 10^5^ cells per well. Cells were infected at 1 × 10^5^ PFU/well (MOI 0.2) with SARS-CoV-2 lineage B [29], 3rd passage and kept for 2 h at 37 °C with 5% CO_2_ in MEM supplemented with 2.5% FBS and 1% penicillin/streptomycin. Cells were rinsed with 1× PBS to remove attached viral particles, and fresh MEM with 10% FBS was added to the cultures. After 48 h, the cell cultures were halted and used for supernatant harvesting [30].

### 2.2. RNA Isolation

RNA samples from culture supernatants were extracted using viral QIAamp Viral RNA Mini Kit (Qiagen, Germantown, MD, USA). Briefly, 350 µL of supernatants were centrifuged at 3000 rpm for 5 min to remove cell debris and transferred to new tubes containing 550 µL of lysis buffer (AVL—provided with the kit) and RNA isolation was performed according to the manufacturer’s instructions. RNA samples were quantitated with Nanodrop (Thermo Fischer Scientific, Waltham, MA, USA).

### 2.3. Direct RNA Sequencing

For RNA sequencing, 9.5 µL of RNA containing ≈50 ng of RNA, from Vero E6 cells supernatant, were used for construction of RNA sequencing libraries with the Nanopore RNA Sequencing kit SQK-RNA002 following the manufacturer’s protocol with cDNA synthesis (Oxford Nanopore Technologies, Oxford Science Park, Oxford, UK). The RNA libraries were loaded and run on a MinION device (Oxford Nanopore Technologies) with flowcell FLO-MIN106 for 40 h and 1.59 million reads were generated. Raw data (fast5 files) were used for basecalling with Guppy (v-5.0.11) in high-accuracy mode.

### 2.4. Assembly

The resulting fastq reads were aligned to the SARS-CoV-2 reference (GISAID ID: EPI_ISL_413016) with minimap2 (v-2.21-r1071) [31]. The resulting sam files were converted to bam files and all reads were sorted and indexed according to the reference coordinates using samtools (v-1.13) [32]. The “index” and “eventalign” modules of nanopolish (v-0.13.3) were used to generate an index of base call reads for the signals measured using the sequencer, and to align events to the reference transcriptome, checking for differences in current that may suggest modifications in the base.

### 2.5. Methylation Analysis

The probability of methylation in DRACH motifs was calculated with m6anet (v-0.1.1-pre) [33], as recommended, (I) by preprocessing the segmented raw signal file with “m6anet-dataprep”, and (II) running m6anet over data using “m6anet-run_inference”.

### 2.6. DRACH Motif Comparison

Comparative analysis and annotation of DRACH motifs [28], identified with m6Anet (v-01.1-pre) [34], among SARS-CoV-2 variants were carried out using Geneious v-10.4 (http://www.geneious.com, accessed on 21 August 2021). Five sequences of each variant isolated and sequenced in Brazil were aligned using the Geneious aligner. For variant Eta only, one sequence from Brazil is available from GISAID (http://www.gisaid.org, accessed on 21 August 2021) that is complete with high coverage and, therefore, samples from the US, France, Spain and Canada were used. The SARS-CoV-2 variants sequences analyzed are deposited in the GISAID database (http://www.gisaid.org, accessed on 21 August 2021) with accession numbers for Alpha (EPI_ISL_1133259, 1133268, 1133267, 1495029, 3316204), Beta (1966629, 1742275, 1966124, 1445171, 1716877), Gamma (3539883, 3539773, 3545813, 3545803, 3540000), Delta (3540039, 3540020, 3540001, 3460250, 3505224), Eta (1583653, 3502618, 3535614, 3490821, 3493947), Lambda (1445272, 3010903, 2928137, 1966094, 2617911) and Zeta (3434818, 2841610, 3506974, 3190295, 1494963).

## 3. Results

### 3.1. Direct RNA Sequencing and Assembly

**Figure 1:**
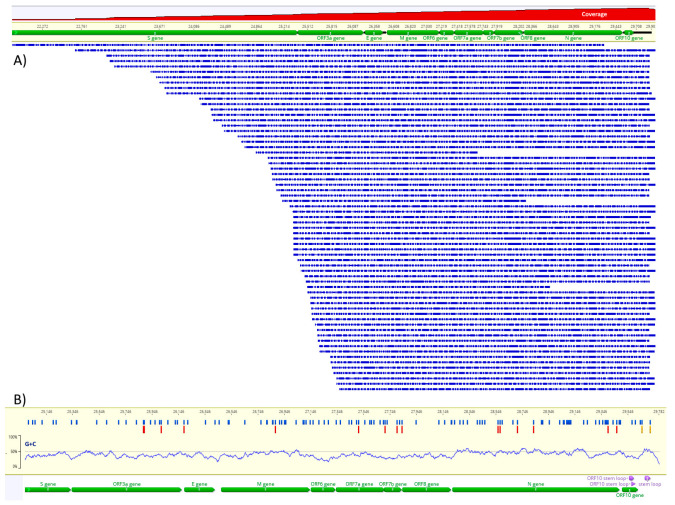
Assembly of Nanopore direct RNA reads to the reference sequence (**A**) and map of m6A methylation probability along the SARS-CoV-2 RNA (**B**). In (**A**), green horizontal bars indicate the ORFs, blue horizontal bars of decreasing size indicate the Nanopore reads and the red area at the top indicates the log scale coverage from 1× to 1600×. In (**B**), blue vertical bars indicate the DRACH motifs, red vertical bars indicate m6A (>50% probability) and the yellow vertical bars indicate two potential m6A with probabilities 0.38 and 0.44 in the 3′-untranslated region. The G + C content is indicated in the plot just above the annotation.

The assembly of the 3′-half of the SARS-CoV-2 RNA genome was obtained by mapping the RNA reads to GISAID ID: EPI_ISL_413016, the strain used for the infection of the Vero E6 cells (Figure 1A). The coverage varied from 30× to 1600× from 5′ to 3′ starting at ORF3a. As the RNA sequencing adapter is ligated to the 3′-ends of RNAs, the coverage is higher as it gets closer to the 3′-end. Several reads reached the “Spike” ORF but were not used for further analysis due to low coverage. The sequencing runs of 40 h were sufficient to obtain around 2000 reads corresponding to the 3′-half of the SARS-CoV-2 genome. The average sequence length was 787 bases and the mode 1350 bases. The global identity to the reference was 90%. The phred scores of the assembled bases are between 20 and 30. ORF N has a substantial coverage because of its proximity to the 3′-end (≈1000×).

### 3.2. Detection of m6A

Direct RNA sequencing reads were used for the detection of m6A using the m6Anet tool, validated by systematic benchmark [35]. This method uses the reference sequence, the basecalled fastq files and the raw fast5 files to identify the DRACH motifs and the raw signal data in fast5 files with corresponding signal alterations associated with m6A to calculate the probability of bona fide methylation [35]. Using this approach, we identified 15 positions within DRACH with ≥50% methylation probability in at least one replicate (Figure 1B). Among these positions, 11 have more than 100× coverage and four positions have >80% methylation probability (Table 1). The nucleocapsid region (N) contains six putative m6A residues and at least 30 DRACH motifs.

### 3.3. DRACH Motif Analysis

**Figure 2:**
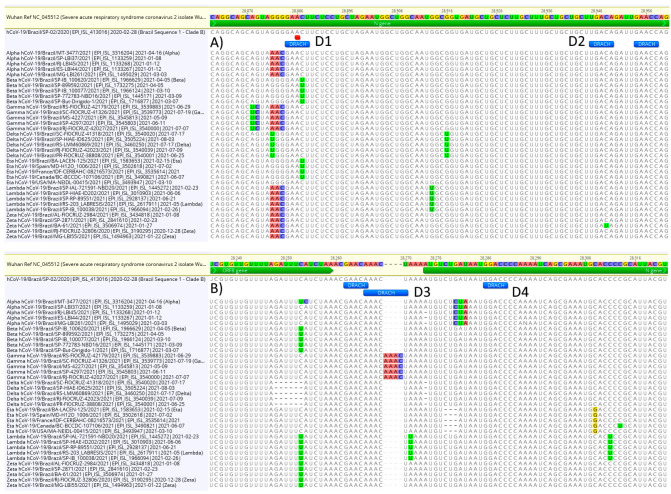
Variability of DRACH sequences among SARS-CoV-2 variants. In (**A**), the C > U change at 28,886 disrupts the m6A (marked red) DRACH motif (D1) in five variant Beta sequences and four variant Eta sequences, while a single variant Zeta has a C > U mutation that disrupts the DRACH motif (D2). In (**B**), a four bases insertion in the intergenic region ORF8/N does not disrupt DRACH (D3), while (D4) shows a fully conserved DRACH.

As the DRACH motif is associated with m6A, we tested if the major SARS-CoV-2 variants, Alpha, Beta, Gamma, Delta, Eta, Lambda and Zeta, isolated in Brazil exhibited mutations within DRACH that differ between the variants. The alignment consisted of the Wuhan reference sequence (GenBank NC_045512), the Brazilian reference (GISAID ID: EPI_ISL_413016) and five sequences of major variants (Material and Methods). DRACH is highly conserved among variants; however, differences can be observed (Figure 2). Five sequences of the variant Beta and four of the variant Eta have a C > U change in the fourth position of DRACH (at position 28,886) that could block methylation at this site (Figure 2A, D1). The methylation probability at this site is 70% and the coverage 871×. Another change in DRACH that could probably interfere with methylation is a C > U at 28,947 (Figure 2A, D2) in a single variant Zeta sequence, although the methylation probability at this site is <50%. Other DRACH variants observed are probably “silent”, such as the four nucleotides insertion in the intergenic region between ORF8 and ORF N in the five sequences of variant Gamma (Figure 2B, D3). The insertion does not change the DRACH sequence.

## 4. Discussion

RNA modification, or epigenomics, is a key factor in viral infections [14]. The methylation of RNA bases, either as the host’s multitargets or viral RNAs, is involved in immunity and associated disease progression [17,36]. Various types of RNA methylation are involved in COVID-19 immunity and novel proposed treatments involve methylase inhibitors [37,38]. The Nanopore direct RNA sequencing offers a unique opportunity for the study of viral epigenomics [22,24]. Direct RNA sequencing is PCR-free, is not sequencing-by-synthesis and, therefore, not affected by PCR bias and synthesis errors. The modified bases are preserved and assessed directly with no need for chemical treatments or antibodies. Direct RNA sequencing of SARS-CoV-2 has been validated using orthogonal methods [27].

In the present study, we show that direct RNA sequencing of SARS-CoV-2 is a valuable tool for the assessment of the full complexity of this viral RNA genome. The identification of m6A was performed in supernatants of SARS-CoV-2-infected Vero E6 cells and, therefore, is enriched in genomic RNAs and not subgenomic RNA [25]. Sequencing was performed without PCR amplification or any other in vitro synthesis (Figure 1).

The mapping of m6A using direct RNA sequencing data was achieved by a comparison of raw and basecalled reads. The m6A pattern identified is consistent with the nucleocapsid region m6A enrichment observed using liquid chromatography-tandem mass spectrometry and methylated RNA immunoprecipitation sequencing (MeRIP-seq) [17]. The probability of m6A, as calculated using the m6Anet method, was >50% and a minimum coverage of 60× in four positions and the recommended 100× coverage in 11 positions in the first experiment [35]. In Figure 1 B, the m6A positions are confirmed by at least one experiment for a ≥50% probability. Positions that are not methylated almost always have probabilities below 5%. Our results suggest that the nucleocapsid region has more methylated sites (Table 1), which is consistent with previous studies using Met-RIP, mass spectrometry (for m6A) and direct RNA sequencing (for 5mC) [17,18,24]. Our m6A pattern is based on sequencing data from two replicates. Although some positions are consistent between two experiments (e.g., 27,764), others vary (e.g., 28,616). The analysis of the output of m6Anet program shows that the positions putatively methylated have a significantly higher probability (>25%) than the putatively non-methylated (<5%), which is consistent with the model training and benchmarking [33]. Therefore, positions that, in at least one experiment, have a ≥50% probability are indicative that a substantial proportion of modified bases, over unmodified, are present in the sample. The m6A detection method employed here uses a model that takes into account the mixture of modified and unmodified RNAs and outputs the m6A-modification probability at any given site for all the DRACH 5-mers represented in the neural network training data and this might, at least in part, explain the variance among experiments as observed with different cell lines [33]. As a future perspective, the m6Anet model can be trained for the SARS-CoV-2 genome, which might adjust the probability values for m6A prediction in these specific RNAs.

As expected, the DRACH pattern is highly conserved among SARS-CoV-2 variants, although, in at least one position, a significant mutation was observed in the nucleocapsid region in variants Beta and Eta (Figure 2) that could negatively affect methylation by disrupting the DRACH motif [28]. The DRACH motif is highly conserved and strongly associated with m6A methylation (in the middle “A”) and, therefore, provides another level for selective pressure on SARS-CoV-2. The significance of this finding needs to be further investigated with comparative infection experiments using different combinations of viral and host cell lineages to determine the impact of DRACH mutations in viral growth. Additionally, differential methylation among the variants must be explored because experimental data with knockdown METTL3 viruses suggest that hypomethylated viral genomes are produced and the infection is significantly reduced [17].

As a future perspective, we are working on a methodology to allow full length, high coverage sequencing of the whole SARS-CoV-2 RNA genome by direct RNA sequencing and, therefore, extend the m6A analysis to ORF1ab, Spike and the 5′-untranslated region to compare the differential methylation among lineages and variants.

## Figures and Tables

**Table 1:** Distribution and sequencing coverage of potential methylated adenosines in SARS-CoV-2 RNA genome. Coverage > 100× is underlined and probability > 80 is in boldface as calculated by m6Anet [34]. After m6Anet analysis, only sites with coverage above 60 were considered. Position numbering according to Wuhan reference sequence (GenBank NC_045512). In Coverage and Probability columns, the results for two technical replicates are shown. The first numbers on these columns represent the experiment shown in Figure 1.

#	RNA Id—ORF	Position	Coverage	Probability
1	EPI_ISL_413016—3a	25,935	65/75	0.5472/0.2749
2	EPI_ISL_413016—3a	25,940	67/61	0.5094/0.5865
3	EPI_ISL_413016—3a	26,070	72/83	0.6659/0.2497
4	EPI_ISL_413016—3a/E	26,241	83/100	0.5480/0.3461
5	EPI_ISL_413016—M	26,933	162/295	0.5102/**0.8204**
6	EPI_ISL_413016—7a	27,562	266/679	**0.8516**/0.3973
7	EPI_ISL_413016—7b	27,764	309/849	**0.8433**/**0.8946**
8	EPI_ISL_413016—7b	27,854	318/839	**0.8761**/**0.8709**
9	EPI_ISL_413016—7b/8	27,892	357/859	0.5770/0.4420
10	EPI_ISL_413016—N	28,616	699/812	**0.8184**/0.4929
11	EPI_ISL_413016—N	28,633	314/364	0.5022/0.4134
12	EPI_ISL_413016—N	28,766	794/817	0.5403/0.4098
13	EPI_ISL_413016—N	28,886	871/835	0.7020/0.2212
14	EPI_ISL_413016—N	29,450	922/803	0.5523/0.2701
15	EPI_ISL_413016—N	29,517	887/783	0.5705/0.3598

## Data Availability

The data presented in this study are available on request from the corresponding author. The data are not publicly available until the manuscript is accepted for publication.
